# UBE2C mediated radiotherapy resistance of head and neck squamous cell carcinoma by regulating oxidative-stress-relative apoptosis

**DOI:** 10.18632/aging.204265

**Published:** 2022-09-05

**Authors:** Yingchun Zhou, Junbin Zhang, Jinglin Gong, Xi Tang, Chengyao Zhang

**Affiliations:** 1 Department of Head and Neck Cancer Center, Chongqing University Cancer Hospital, Chongqing 400030, China; 2 Chongqing Key Laboratory of Translational Research for Cancer Metastasis and Individualized Treatment, Chongqing University Cancer Hospital, Chongqing 400030, China

**Keywords:** UBE2C, oxidative stress, HNSCC, radiotherapy resistance

## Abstract

Purpose: Radiotherapy resistance is the main obstacle in the effective treatment of advanced head and neck squamous cell carcinoma (HNSCC). Increasing scientific opinions present that ubiquitin-conjugating enzyme E2C (UBE2C) might be a target gene acting as an oncogene.

Method: TCGA database was used to analyze the expression of UBE2C in HNSCC patients, and the relationship between UBE2C expression and prognosis. Western blot and RT-PCR were used to assess UBE2C expression before and after radiation. Then, cell viability experiment and colony formation were used to evaluate proliferation after 2 Gy radiation. Cell viability experiment, migration, and invasion were evaluated in the condition of UBE2C knock-down. Western blot and RT-PCR were used to assess the expression of apoptosis and ROS relative gene expression. Then, the xenograft model was used to evaluate the efficacy of radiation combined with UBE2C suppression.

Result: The expression of UBE2C was high in tumors of patients with HNSCC and relatives with poor prognoses. Si-UBE2C cells showed proliferation inhibited and apoptosis enhanced after radiation. Furthermore, the mechanism of UBE2C in HNSCC radioresistance was explored. We performed RT-PCR to find the 4-HNE, which increases oxidative-stress-relative apoptosis in Si-UBE2C cells after radiation.

Conclusions: Through the RT-PCR, WB, cell viability experiment, migration, invasion, and *in vivo* experiment, UBE2C was confirmed to downregulate oxidative-stress-relative apoptosis induced by radiation and promote the development of malignant tumor cells.

## INTRODUCTION

As the most common cancer worldwide, according to the data published by WHO in 2020, HNSCC patients still face many challenges: poor outcome of advanced HNSCC, radiotherapy resistance, recurrence, poor prognosis, etc. [1]. Therapeutic strategies, such as neoadjuvant immunotherapy plus radiotherapy [2], have improved significantly in recent years, but the prognosis of HNSCC remains poor, with about 40-50% 5-year survival rate [3]. And it’s important to discover a new biomarker that can help the diagnosis and radiotherapy difficulty of HNSCC [4].

As a vital member of the E2 family, ubiquitin-conjugating enzyme E2C (UBE2C) plays a major role in cell cycle progression by interacting with the anaphase-promoting complex/cyclostome (APC/C) [5]. And UBE2C has been increasingly researched and recognized as an important marker overexpressed in many malignant tumors, exceeding the development as an oncogene and predicting the poor prognosis of patients [6].

Previous studies have reported that UBE2C expression levels were predictive of a higher rate of pathological complete response rate in luminal A patients with tumor relapse within five years of endocrine therapy [7]. However, the overexpression of UBE2C led to substantial inhibition of colony formation of HNSCC [8], and a novel insight has identified that UB2EC may become a main driver of tumorigenesis and an effective way to sensitize cervical cancer cells to radiation [9]. The precise molecular mechanisms by which cells are sensitized to radiation are unclear and need further investigation.

## RESULTS

### UBE2C is over-expressed in radiotherapy-induced CAL27 and associated with a poor prognosis

**Figure 1:**
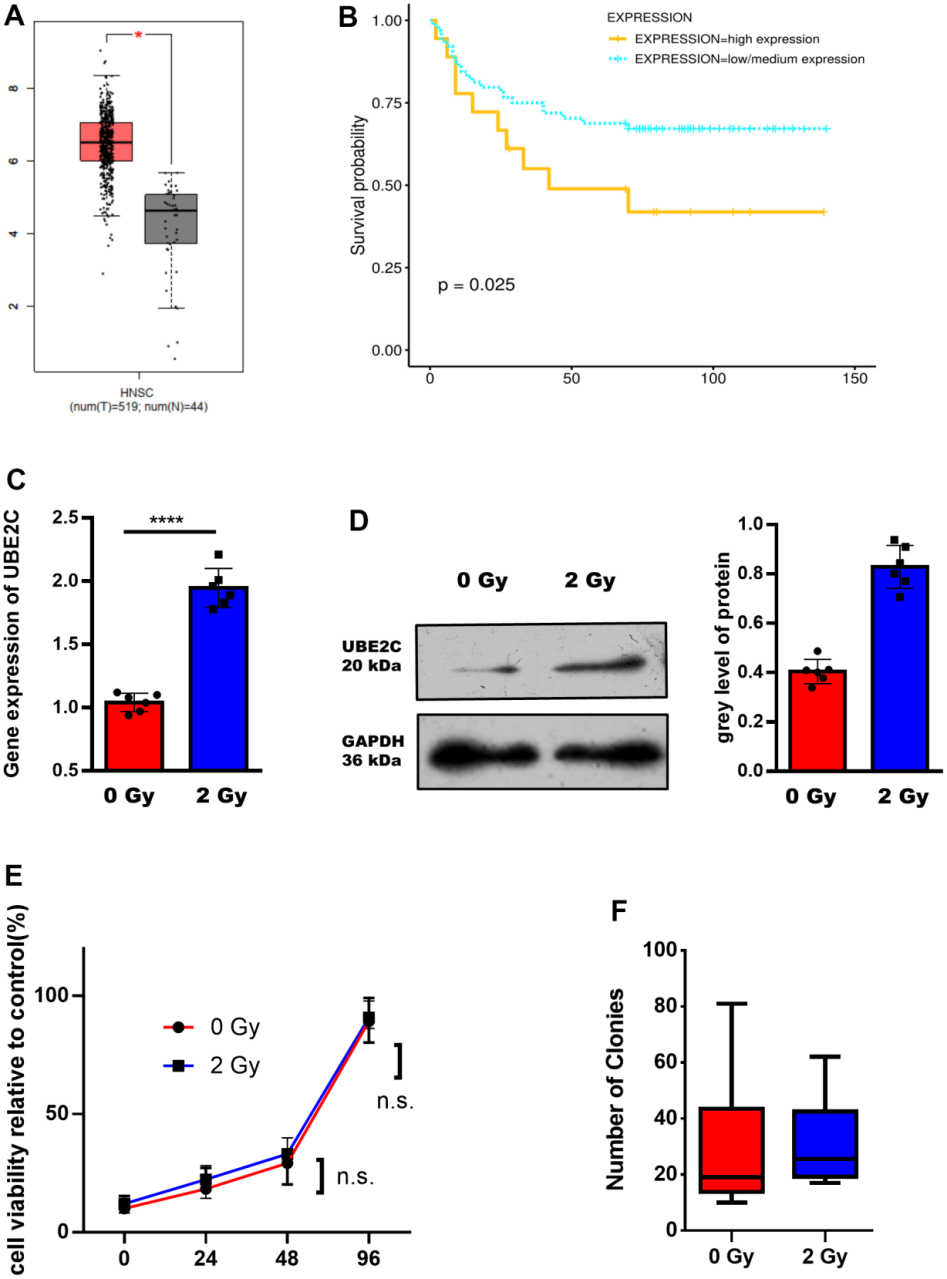
**UBE2C is over-expressed in HNSCC after radiation and associated with the poor prognosis.** (**A**) The expression of UBE2C was different in different subclasses (Tumor group, N=519. Normal group, N=44.). (**B**) The different survival probability between the high expression and low/medium expression of UBE2C were shown (p=0.025). (**C**) RT-PCR result of UBE2C gene expression in CAL27 after 0/2 Gy radiation. (**D**) Western blot result of UBE2C protein expression in CAL27 after 0/2 Gy radiation. (**E**) Cell viability of CAL27 after 0/2 Gy radiation. (**F**) Cell colony formation assay of CAL27 after 0/2 Gy radiation. *P < 0.05, **P < 0.01, ***P < 0.001 versus the control.

By analyzing the UBE2C expression in HNSCC based, we can find a significantly higher expression of UBE2C in the HNSCC compared with the tumor-side group (Figure 1A). The result of numerical matched condition analysis was shown in Supplementary Figure 1. Next, we performed the overall survival (OS) analysis by Kaplan-Meier-plotter. The chart showed that patients with high levels of UBE2C had worse clinical outcomes (Figure 1B). Therefore, we deduced that UBE2C might upgrade the development of HNSCC, and was expected to be a potential indicator for the prognosis of HNSCC. In consideration of the UBE2C overexpression in HNSCC and its critical role in tumor progression, we hypothesized that UBE2C could regulate the radiotherapy sensitivity of human HNSCC. To test the hypothesis, we first focused on the UBE2C expression in the CAL27 cell line with or without radiotherapy. UBE2C mRNA and protein upregulation after 2 Gy radiation was confirmed using qRT-PCR and Western blotting (Figure 1C, 1D). Furthermore, viability assay (Figure 1E) and clone formation (Figure 1F) results showed no difference in CAL27 received 0 Gy and 2 Gy irradiation.

### Knocking-down of UBE2C suppressed the radiotherapy resistance of HNSCC cells

**Figure 2:**
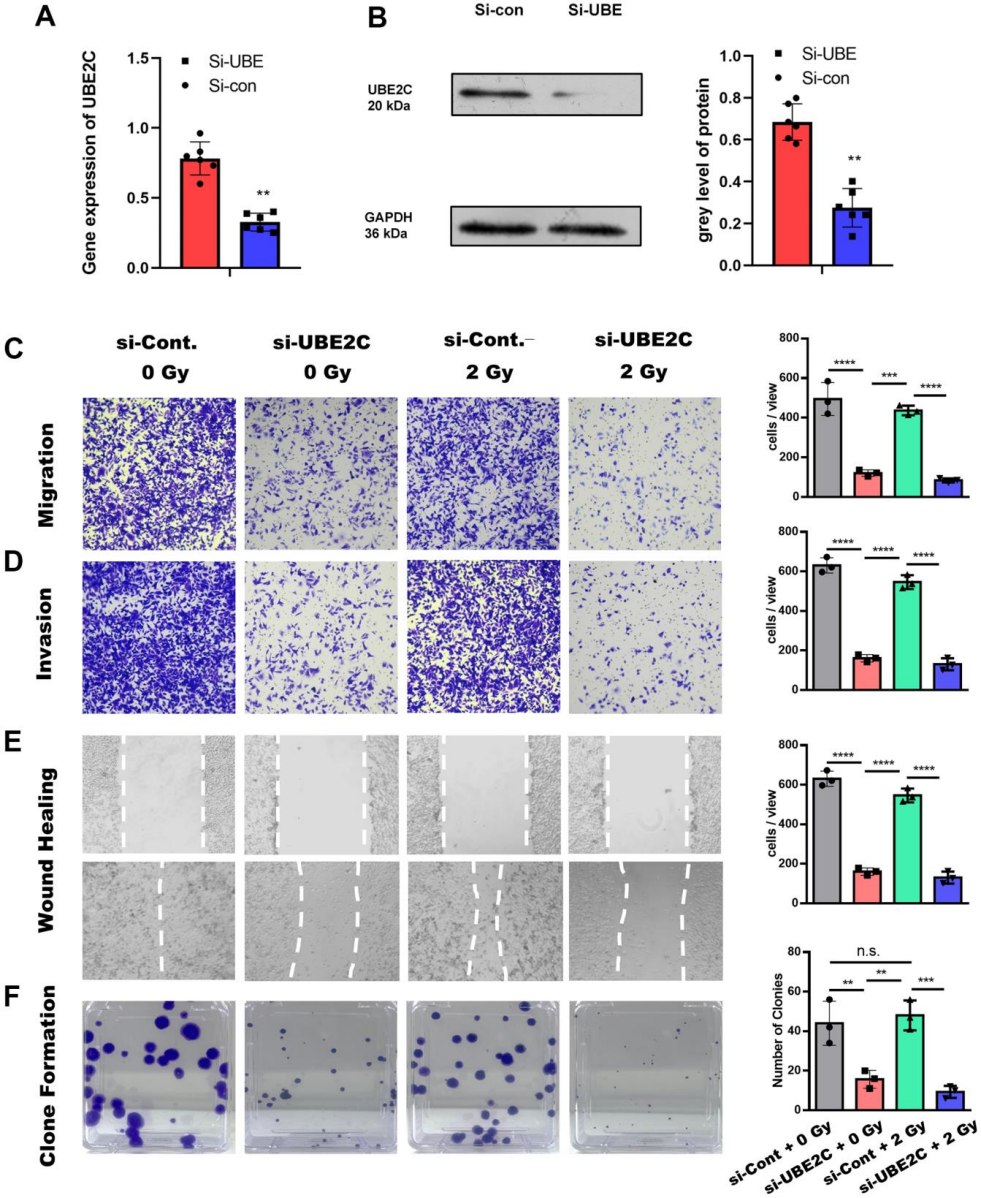
**The knocking-down of UBE2C sensitized CAL27 cell to radiation *in vitro*.** (**A**) RT-PCR results of UBE2C gene expression in CAL27 after Si-UBE/Con Lentivirus transfection. (**B**) Western blot results of UBE2C protein expression in CAL27 after Si-UBE/Con Lentivirus transfection. (**C**) Transwell (without matrigel) results of Si-UBE/Con CAL27 cells under 0/2 Gy radiation after 24h. (**D**) Transwell (with matrigel) results of Si-UBE/Con CAL27 cells under 0/2 Gy radiation after 24h. (**E**) Wound healing results of Si-UBE/Con CAL27 cells under 0/2 Gy radiation after 48h. (**F**) Clone formation results of Si-UBE/Con CAL27 cells under 0/2 Gy radiation after 14d. *P < 0.05, **P < 0.01, ***P < 0.001 versus the control.

To verify the gene’s function, we knocked down the UBE2C using lentiviral-mediated siRNA in CAL27 cell (Si-UBE2C) to check the role of UBE2C in tumorigenesis. The RT-PCR tests and the western-bolt tests demonstrated the common result that the gene expression and protein level of Si-UBE was significantly lower than the Si-con (Cal cell with lentiviral-mediated normal control SiRNA) (Figure 2A, 2B). We next attempted to confirm the effect of UBE2C on the migration and invasion of CAL27 cells with or without radiotherapy. Transwell and scratch wound healing assays were performed to evaluate the response of Si-UBE or Si-con to 0 or 2 Gy irradiation. Regarding invasive ability, transwell assay results showed that the Si-UBE cells in both 0 Gy and 2 Gy groups were significantly less invasive than that in the Si- con cells group (Figure 2C, 2D, P<0.05). The Si-con cells with 0 or 2 Gy treatment showed a smaller scratch wound at 24 h than that in the Si-UBE group (Figure 2E, P<0.05). The cells Si-UBE2C decreased in number and proliferated lower in comparison with Si-con (Figure 2F).

### UBE2C conferred radiotherapy resistance of HNSCC by regulating oxidative-stress-induced apoptosis through 4-HNE signaling

**Figure 3:**
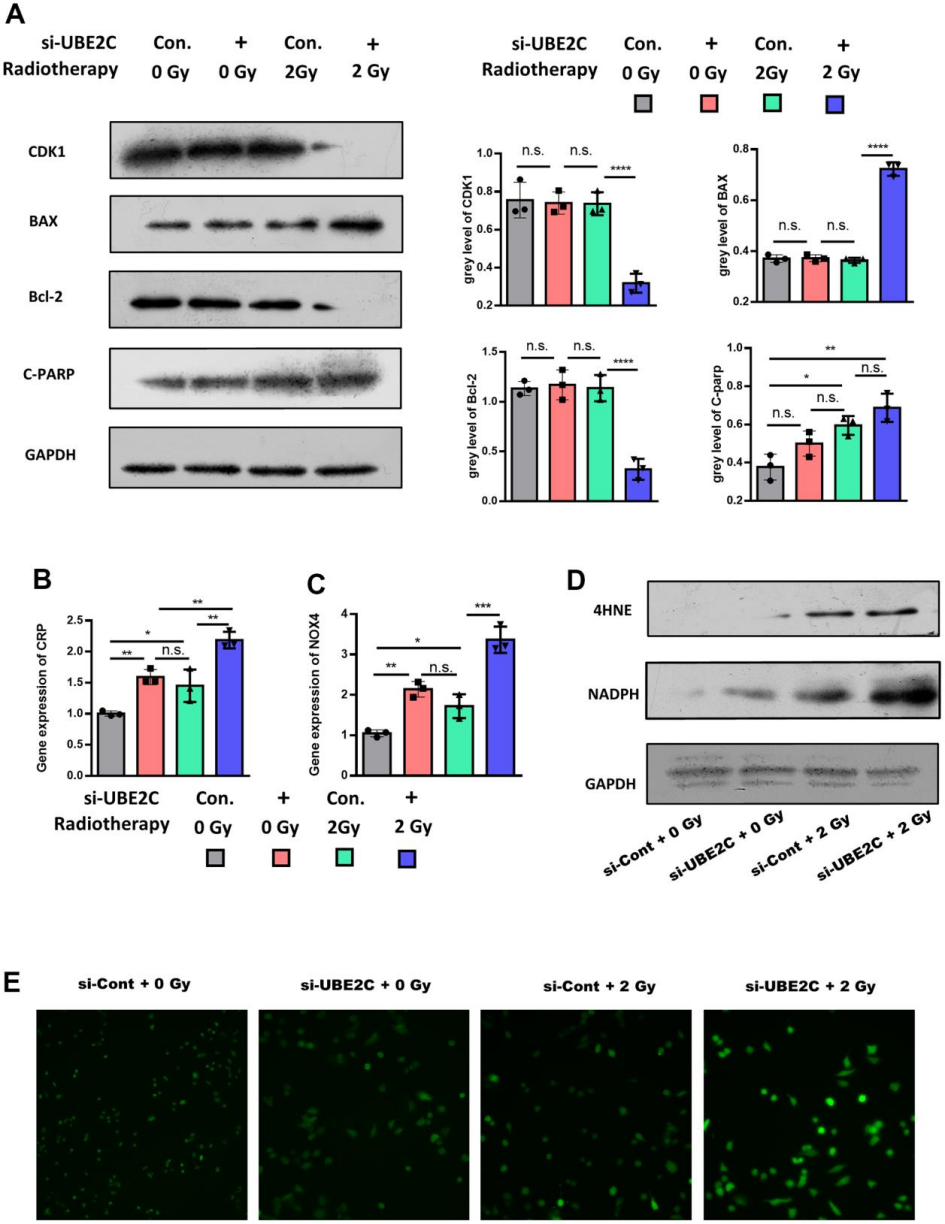
**UBE2C confers radiotherapy resistance of HNSCC by regulating oxidative-stress-induced apoptosis through 4-HNE signaling.** (**A**) Western blot results of proliferation relative protein (CDK1), anti-apoptosis relative protein (Bcl-2) and apoptosis relative protein (C-PARP, BAX) in Si-UBE2C/Cont CAL27 cells under 0/2 Gy radiation. (**B**) RT-PCR results of CRP gene expression in Si-UBE2C/Cont CAL27 cells under 0/2 Gy radiation after 24h. (**C**) RT-PCR results of NOX4 gene expression in Si-UBE2C/Cont CAL27 cells under 0/2 Gy radiation after 24h. (**D**) Western blot results of oxidative stress relative protein (4-HNE and NADPH) in Si-UBE2C/Cont CAL27 cells under 0/2 Gy radiation. (**E**) ROS level in Si-UBE2C/Cont CAL27 cells under 0/2 Gy radiation. *P < 0.05, **P < 0.01, ***P < 0.001 versus the control.

Increasing evidence suggests that oxidative-stress-induced apoptosis involves resistance to anticancer treatments. Next, we screened apoptosis markers, including BAX, Bcl-2, and C-PARP. The Western blot test showed that cyclin-dependent kinases (CDK1), a cell division associated protein, was lower expressed in si-UBE2C, but the apoptosis-associated factor, Bax, was higher expressed [6]. The protein level of C-PARP indicated that cell apoptosis was increased after the knockdown of UBE2C under 2 Gy irradiation (Figure 3A) [10]. And Bcl-2, a protein that suppresses apoptosis, was decreased in Si-UBE with 2 Gy irradiation group. The above results demonstrated that silencing UBE2C significantly sensitized CAL27 cells to radiation.

The mechanism of UBE2C promoting the progression of HNSCC was unclear [9]. Thus, we conducted RT-PCR to explore the downstream of UBE2C by comparing the Si-UBE and Si-con. The RT-PCR result showed that the oxidative stress relative gene, including NOX4 [11] and CRP [12], were significantly up-regulated in Si-UBE after 2 Gy radiation (Figure 3B, 3C), as well as the oxidative stress biomarker 4-HNE (Figure 3D).

### Silencing UBE2C sensitized CAL27 to radiation *in vivo*

**Figure 4:**
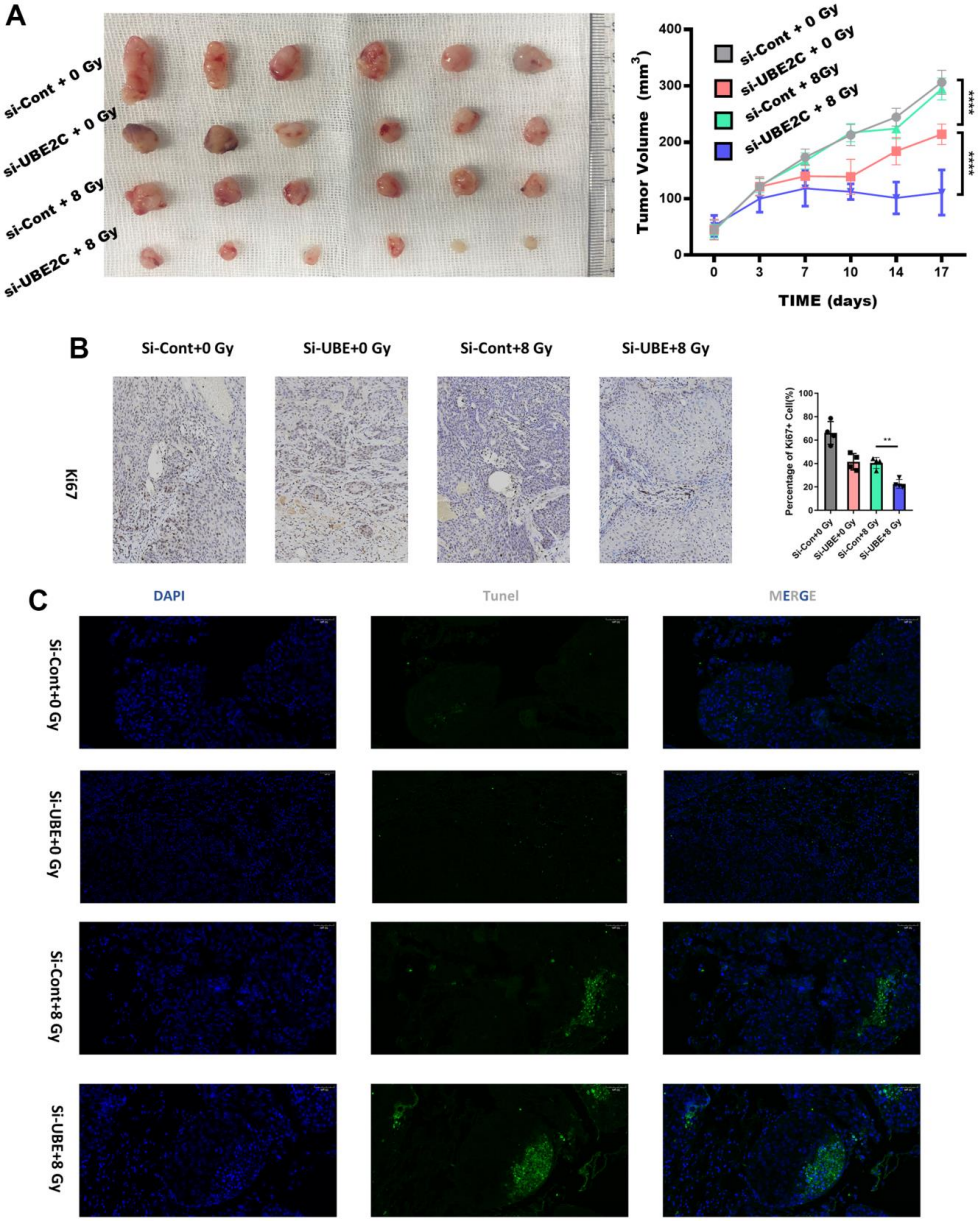
**Silencing UBE2C sensitized CAL27 to radiation *in vivo*.** (**A**) Representative images of subcutaneous tumors after treatment. The curves describe the average tumor volume *in vivo* of four different groups at different observing time. (**B**) Representative images of tumors from xenografts using immunohistochemical staining against Ki67. Magnification: ×200. (**C**) The percentage of TUNEL-positive cells was assessed in formalin-fixed paraffin embedding sections of tumors in each group. Magnification: ×200. *P < 0.05, **P < 0.01, ***P < 0.001 versus the control.

In order to evaluate the radio-resistance induced by UBE2C in an *in vivo* animal model, we established a CAL27 xenograft mice model by subcutaneously implanting Si-UBE2C/Si-con cells (2×10^6^ cells) into balb/c nude mice. These mice were subjected to radiotherapy when their tumor volume reached approximately 50 mm^3^. According to Figure 4A, tumor volume in the Si-UBE2C+8 Gy group was smaller than that in the Si-con+8 Gy group. Radiotherapy decreased tumor growth rate in both Si-UBE2C and Si-con groups. And the IHC test showed the Ki67 distributed significantly less in Si-UBE2C+8 Gy group (Figure 4B). The Tunel apoptotic tests showed the green faculae were in the widest distribution in the Si-UBE2C+8 Gy group (Figure 4B and Supplementary Figure 2). Above all, the results demonstrated that UBE2C inhibited the biological function of HNSCC cells.

## DISCUSSION

Research has presented that UBE2C participates in many human malignancies, including ovarian cancer, gastric cancer, and head and neck cell carcinoma [8]. Now we first confirmed that the UBE2C was overexpressed in HNSCC, especially in radiotherapy-resistant HNSCC, employing the public database, TCGA. And then, we set up the si-UBE2C cell lines and compared them with the Si-Con cell line to verify the migration, invasion, and proliferation ability after radiotherapy. Besides, Kaplan Meier-plotter analysis was conducted to confirm the correlation between the UBE2C with the poor prognosis of HNSCC.

Ubiquitin-conjugating enzyme E2 C (UBE2C), one important member of the E2 family in the ubiquitin-proteasome system (UPS), has been reported to be involved in cell cycle regulation [13]. And it is an essential protein required for the destruction of mitotic cyclins and fatal for cell progression, which was correlated with cancer development [14, 15]. In the field of breast cancer, some research found that UBE2C was regulated by estrogen-independent growth, which has been proved to be a tumorigenic factor for ER-positive HER2 negative BC cells [6]. Associated with the previous study, we designed the experiment to clarify further.

Recent research has reported that UBE2C played an important role in the radiotherapy of malignant tumors [9], whose downregulation might increase the sensitivity of tumor cells. To prove the opinion, we established the Si-UBE2C cell line, and in the cell line, UBE2C was low expressed. So we deduced the UBE2C might be a potential factor regulating the radio-resistance via ROS signaling. Furthermore, UBE2C Si-RNA downregulation cell lines were built to verify that after the under-expression, the proliferation and cell division were suppressed, and the apoptosis was enhanced. Thus, UBE2C might be a relevant gene affecting radiotherapy resistance.

However, the further molecular mechanism of UBE2C-regulating radio-resistance is still unclear. Under the influence of UBE2C down-regulation, the expression levels of those proliferation and anti-apoptosis associated genes, such as CDK1 and Bcl-2, were inhibited. Adversely, the apoptosis genes, such as Bax and C-PARP, were upregulated. According to former research, approximately 80% of radiation-induced apoptosis is caused by indirect damage via ROS production [16]. Consequently, increased oxidative stress has been observed after irradiation, which plays an important role in effective radiotherapy [17, 18]. According to RT-PCR results, oxidative-stress-relative genes were significantly increased at mRNA expression level after UBE2C inhibition. This phenomenon indicated that UBE2C might increase radio-resistance by suppressing oxidative-stress-relative apoptosis in CAL27 cells. Moreover, 4HNE, a protein increasing oxidative stress, was found to be up-regulated after UBE2C silencing. Despise the progression we made in this research, the mechanism of how UBE2C regulated the expression of 4-HNE was still unclear. which might be the next step of our future research.

In summary, our study uncovered a critical role of UBE2C in regulating radiotherapy resistance to cancer. Our study demonstrated for the first time that low expression of UBE2C might sensitize HNSCC to radiotherapy, possibly through increasing 4HNE expression and the subsequent activation of oxidative stress. The connection between radiotherapy resistance development and UBE2C shed new light on our understanding of the treatment response modifications of cancer.

## MATERIALS AND METHODS

### Cell lines and cell culture

The HNSCC cell line, CAL27, was purchased from the American Type Culture Collection (ATCC). All the cells were cultured in PRIM-1640 with 10% fetal bovine serum (FBS) (GIBICO) and 1% penicillin and streptomycin (Beyotime Biotechnology, Shanghai, China) and the cells were cultured at 37° C, 5%CO2.

### Transfection

The lentiviral-mediated siRNA interference was used to knock down UBE2C in the CAL27 radiotherapy resistance cell line. The siRNA and si-Con (the negative control) were purchased from the Genenchem Company (Shanghai, China). The target sequences of UBE2C follows: 5’-ccTGCAAGAAACCTACTCAAA-3’ and 5’-TTTGAGTAGGTTTCTTGCAGG-3’. And the stable transfection passage cell line was purified with 2 μg/ml Puromycin for 72 h.

### Cell colony formation assay

Cells were plated into T25 flasks with a concentration of 2 x 10^3^ per well, and every 3 d the medium was refreshed. Then cells were exposed to 0 or 2 Gy doses of irradiation. After 12 d, the cells were treated with 4% polyformaldehyde, and after being washed twice with PBS, the cells were added to a 0.5% crystal violet staining solution (Yeasen, Shanghai, China) [10]. ImageJ software (version 1.8.0) was used to count the clone numbers.

### Wound healing

Si-Cont and Si-UBE2C cells were seeded in a 6-well plate (5×10^5^ cells per well). 48 h later, the wounds were scratched using a sterile pipette tip. The remaining cells were washed twice in serum-free culture media and exposed to 0 or 2 Gy doses of irradiation, and wound closure was then observed. Images at 0 and 48 h were captured using a phase-contrast microscope (magnification, ×100), and the percent of wound closure was calculated: [(Ai-At)/Ai] × 100, where Ai represents the initial area of the wound at 0 h and At represents the area of the wound after 24 h.

### Invasion and migration

To evaluate the invasive capacity, Si-Cont and Si-UBE2C cells were suspended in 200 μl serum-free medium, loaded into an 8-μm pore chamber, and inserted into 24-well cell culture plates. DMEM was added to the upper chamber with 10% matrigel at the bottom to evaluate invasion ability of cells, and 10% FBS was added as a chemotactic agent. Cells were exposed to 0 or 2 Gy doses of irradiation and incubated at 37° C and 5% CO2 for 24 h. After incubation, the remaining non-invasive cells were removed from the upper chamber with a cotton swab. The cells that invaded the lower membrane surface were subject to 30-min fixation with 10% paraformaldehyde at room temperature, washed with PBS, and then stained with 0.1% crystal violet. The cells were then viewed under the GX53 inverted microscope. Five fields were randomly selected to acquire images, and ImageJ software (version 1.8.0) was used to count the migratory units.

### Cell viability assay

The cells were plated into 96-well plates (3×10^3^ per well) repeated five wells for every group and exposed to 0 or 2 Gy doses of irradiation. And the cells were continuously cultured till the set examination time point of 0h, 24h, 48h, and 96h. The Cell Counting Kit-8 (Beyotime Biotechnology, Shanghai, China) was used to conduct the assay. And the spectrophotometric data were analyzed with GraphPad 8.0.

### Quantitative real-time PCR

The RNA was collected by using the Takara MiniBEST Universal RNA Extraction Kit. The total mRNA was extracted from HNSCC cell lines. The Reverse Transcription Mix and PrimeScript RT Master Mix were also purchased from the Takara company, and the experimental protocols followed the instruction. The purity and concentration of the RNA were assessed by using a NanoDrop 2000/2000C spectrophotometer at wavelengths of 260/280 nm. A PrimeScript™ RT Reagent Kit (TaKaRa Biotechnology) was used to reverse transcribe RNA into cDNA. The resultant cDNA was used as a template in a TB Green® Premix Ex TaqTM Kit (TaKaRa Biotechnology) master mix, and qPCR reactions were performed on a StepOnePlusTM Real-Time PCR System. The homo UBE2C forward primer was 5’-GACCTGAGGTATAAGCTCTCGC-3’ and the reverse primer 5’-TTACCCTGGGTGTCCACGTT-3’. The homo NOX4 forward primer was 5’-CAGATGTTGGGGCTAGGATTG-3’ and the reverse primer 5’-GAGTGTTCGGCACATGGGTA-3’. The homo NOX4 forward primer was 5’-ATTTCCGTGGCTGGTACATTAG-3’ and the reverse primer 5’-ATGGCTGGTTGTTCGTCATCC-3’. The GAPDH forward primer is 5’- GGAGCGAGATCCCTCCAAAAT-3’ and the reverse is 5’- GGCTGTTGTCATACTTCTCATGG-3’.

### Western blot

The protein was extracted from cells using protein lysis, and the concentration was detected using the BCA method. Then, 20 μg protein sample from every group was added into a gel well of 10% SDS-PAGE, separated at 110 V for 90 min, and transferred to PVDF membranes at 90V for 90 min. At room temperature, the PVDF membrane was blocked in 5% bovine serum albumin (BSA) for 2h. Then, the primary antibody solutions were used to react with the membrane at 4° C overnight. The UBE2C, CDK1, BAX, C-CAS3, C-PARP, and GAPDH Antibody were all bought from the Abcam company, and the secondary antibodies were brought from Biotechnology. After the reaction with secondary antibodies for 2h, the membrane was developed with Pierce™ ECL in ChemiDoc MP (Bio-Rad, Hercules, CA, USA).

### Tumor xenografts

All SPF 8-week-old BALB/c mice were injected with 1x 10^6^ Si-Cont and Si-UBE2C cells and observed for one week. From the first day of the new week, the mice were divided into four groups. When the tumor volumes reached 50 mm^3^, the animals were treated with local irradiation of 8 Gy once a week. After 17 days, the mice of every group were sacrificed and the tumor was measured in weight. And the tumor samples were fixed in 4% polyformaldehyde for the next experiment. The HE experimental pathology and TUNEL experiment were conducted following the standard instruction.

### Bio-informational analysis

The gene expression of UBE2C in HNSCC and normal tissue were evaluated through the TCGA database. And we get the analysis result of survival comparison by using the Kaplan Meier-plotter method through the TCGA data.

### Statistical analysis

Relevant experimental data are presented as the mean±SD. We conducted the statistical analysis using one-way analysis of variance (ANOVA) and Tukey’s multiple comparison test. The student t-test was used to evaluate the difference between the two groups. P<0.05 was statistically considered significant. All statistical analyses were performed using GraphPad Prism.

## Supplementary Material

Supplementary Figures
