# Supplementary material for: UBE2C mediated radiotherapy resistance of head and neck squamous cell carcinoma by regulating oxidative-stress-relative apoptosis
Source: Aging (Albany NY). 2022 Sep 5;14(17):7003–13. doi: 10.18632/aging.204265 (PMC9512496; doi:10.18632/aging.204265)
Supplement: Supplementary Figures [file aging-14-204265-s001.pdf]

SUPPLEMENTARY FIGURES

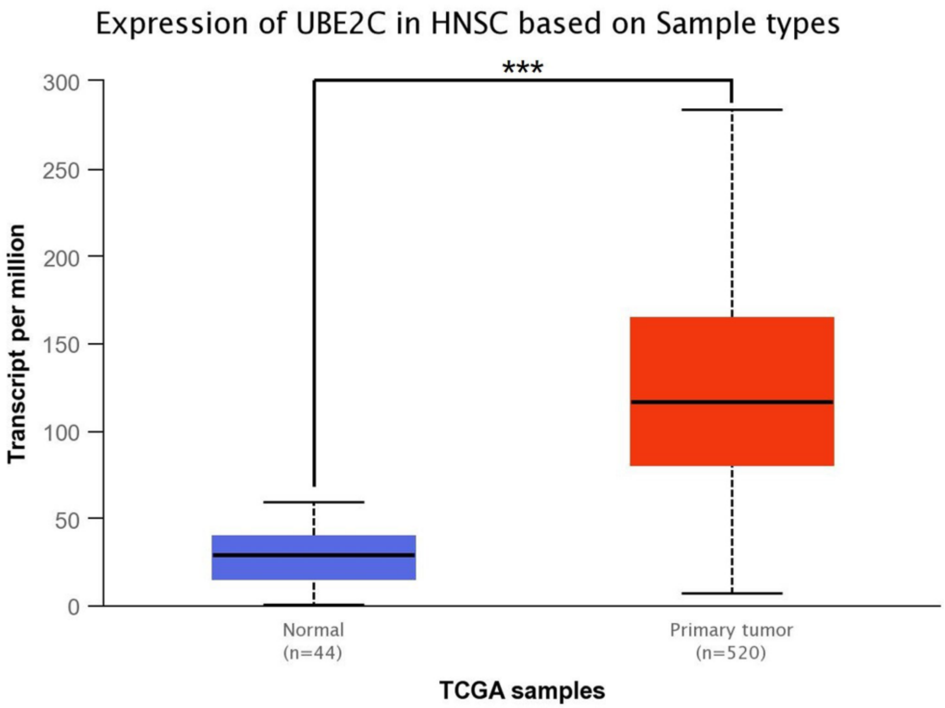

Supplementary Figure 1. The expression of UBE2C by performed numerical matched condition analysis (Tumor group, N=520. Normal group, N=44.). ***P<0.001 versus the normal group. (P=1.62e-12).

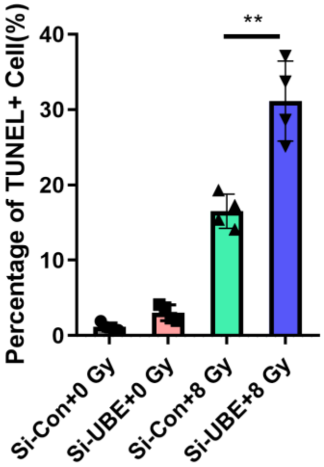

Supplementary Figure 2. The statistical graph of TUNEL. *P < 0.05, **P < 0.01, ***P < 0.001 versus the control.
